# Lipopolysaccharide‐Induced Bone Loss in Rodent Models: A Systematic Review and Meta‐Analysis

**DOI:** 10.1002/jbmr.4740

**Published:** 2022-12-05

**Authors:** Kirsten N. Bott, Evelyn Feldman, Russell J. de Souza, Elena M. Comelli, Panagiota Klentrou, Sandra J. Peters, Wendy E. Ward

**Affiliations:** ^1^ Department of Kinesiology Brock University St. Catharines ON Canada; ^2^ Centre for Bone and Muscle Health Brock University St. Catharines ON Canada; ^3^ Lakehead University Library, Lakehead University Thunder Bay ON Canada; ^4^ Department of Health Research Methods, Evidence, and Impact, Faculty of Health Sciences McMaster University Hamilton ON Canada; ^5^ Population Health Research Institute, Hamilton Health Sciences Corporation Hamilton ON Canada; ^6^ Department of Nutritional Sciences University of Toronto Toronto ON Canada; ^7^ Joannah and Brian Lawson Centre for Child Nutrition University of Toronto Toronto ON Canada; ^8^ Department of Health Sciences Brock University St. Catharines ON Canada

**Keywords:** MICRO‐COMPUTED TOMOGRAPHY, DUAL‐ENERGY X‐RAY ABSORPTIOMETRY (DXA), BONE HISTOMORPHOMETRY, BIOCHEMICAL MARKERS OF BONE TURNOVER, PRECLINICAL STUDIES

## Abstract

Osteoporosis has traditionally been characterized by underlying endocrine mechanisms, though evidence indicates a role of inflammation in its pathophysiology. Lipopolysaccharide (LPS), a component of gram‐negative bacteria that reside in the intestines, can be released into circulation and stimulate the immune system, upregulating bone resorption. Exogenous LPS is used in rodent models to study the effect of systemic inflammation on bone, and to date a variety of different doses, routes, and durations of LPS administration have been used. The study objective was to determine whether systemic administration of LPS induced inflammatory bone loss in rodent models. A systematic search of Medline and four other databases resulted in a total of 110 studies that met the inclusion criteria. Pooled standardized mean differences (SMDs) and corresponding 95% confidence intervals (CI) with a random‐effects meta‐analyses were used for bone volume fraction (BV/TV) and volumetric bone mineral density (vBMD). Heterogeneity was quantified using the *I* ^2^ statistic. Shorter‐term (<2 weeks) and longer‐term (>2 weeks) LPS interventions were analyzed separately because of intractable study design differences. BV/TV was significantly reduced in both shorter‐term (SMD = −3.79%, 95% CI [−4.20, −3.38], *I* ^2^ 62%; *p* < 0.01) and longer‐term (SMD = −1.50%, 95% CI [−2.00, −1.00], *I* ^2^ 78%; *p* < 0.01) studies. vBMD was also reduced in both shorter‐term (SMD = −3.11%, 95% CI [−3.78, −2.44]; *I* ^2^ 72%; *p* < 0.01) and longer‐term (SMD = −3.49%, 95% CI [−4.94, −2.04], *I* ^2^ 82%; *p* < 0.01) studies. In both groups, regardless of duration, LPS negatively impacted trabecular bone structure but not cortical bone structure, and an upregulation in bone resorption demonstrated by bone cell staining and serum biomarkers was reported. This suggests systemically delivered exogenous LPS in rodents is a viable model for studying inflammatory bone loss, particularly in trabecular bone. © 2022 The Authors. *Journal of Bone and Mineral Research* published by Wiley Periodicals LLC on behalf of American Society for Bone and Mineral Research (ASBMR).

## Introduction

Osteoporosis is a disease characterized by low bone mineral density (BMD) and weakened bone structure and is estimated to affect more than 500 million people worldwide.^(^ ^1^ ^)^ Approximately one in three women and one in five men will suffer an osteoporotic fracture in their lifetime.^(^ ^2^ ^)^ Fragility fractures, resulting from low trauma (e.g., a fall from standing height or less), can result in decreased quality of life, increased risk of future fractures, morbidity, mortality and impose a significant financial burden on the healthcare system.^(^ ^3^, ^4^ ^)^ An estimated 158 million individuals worldwide were at a high risk of a fragility fracture in 2010 and this is expected to double by 2040^(^ ^5^ ^)^; therefore, research investigating intervention strategies can have a major public health impact.

Human studies investigating approaches to preventing fragility fractures are limited by the duration required to observe a response, as well as the available analysis techniques that can be performed due to limitations associated with patient radiation exposure. Preclinical rodent models are useful for studying loss of bone mineral and structure because they mimic skeletal changes observed in humans over their lifespan and provide an accelerated model for studying bone outcomes.^(^ ^6^ ^)^ These models provide useful information about disease pathophysiology and for the development of novel treatment interventions.

Traditionally, the pathophysiology of osteoporosis has emphasized endocrine mechanisms; however, more recent evidence suggests that inflammation is also a contributor to this disease.^(^ ^7^ ^)^ Bone is continually remodeled throughout the lifespan by a tightly regulated process mediated by bone‐forming osteoblasts and bone‐resorbing osteoclasts. However, this becomes dysregulated in inflammatory environments, disproportionately favoring bone resorption.^(^ ^8^ ^)^ In older women, inflammatory markers interleukin (IL)‐6 and tumor necrosis factor (TNF) have been associated with a higher risk of hip fracture.^(^ ^9^ ^)^ In men, the risk of hip or vertebral fracture was also positively associated with IL‐6 and TNF‐α.^(^ ^10^ ^)^ Furthermore, diseases associated with an underlying inflammatory pathophysiology such as rheumatoid arthritis,^(^ ^11^ ^)^ inflammatory bowel disease,^(^ ^12^ ^)^ pancreatitis,^(^ ^13^ ^)^ nonalcoholic fatty liver disease,^(^ ^14^ ^)^ and cardiovascular disease,^(^ ^15^ ^)^ are often accompanied by low BMD. Aside from the associated underlying inflammatory pathophysiology, other contributing factors include mobility impairment, nutrient malabsorption, and hormonal alterations that may contribute to a high risk of developing secondary osteoporosis with the aforementioned diseases. There is strong evidence for the role of inflammatory cytokines in osteoporotic bone loss,^(^ ^16^ ^)^ but there is no consensus on a preclinical rodent model of systemic inflammation that induces a deterioration of bone tissue that mirrors osteoporosis.

One approach to inducing systemic inflammation is with lipopolysaccharide (LPS), a component of the outer membrane of gram‐negative bacteria found in the intestines or in the diet that can enter circulation. In humans, the translocation of LPS into systemic circulation has been linked with multiple disease states; at high concentrations LPS induces sepsis, whereas lower concentrations trigger low‐grade inflammation.^(^ ^17^ ^)^ Additionally, there is an increase in gut permeability with aging, leading to elevated circulating LPS thought to contribute to age‐related inflammation.^(^ ^18^, ^19^ ^)^ LPS is also a potent inducer of bone resorption by upregulating the differentiation and activity of osteoclasts^(^ ^20^ ^)^ and inhibiting bone‐forming osteoblasts.^(^ ^21^ ^)^ Systemic LPS stimulates the immune system to release pro‐inflammatory cytokines (e.g., IL‐1, IL‐6, TNF‐α) that can further exacerbate the altered bone metabolism that favors bone resorption.^(^ ^22^ ^)^ LPS also stimulates granulocyte colony‐stimulating factor,^(^ ^23^, ^24^ ^)^ which induces bone resorption while inhibiting bone formation.^(^ ^25^ ^)^ This dysregulated metabolism can alter bone structure and BMD. Although ovariectomy in rodent models mimics the estrogen deficiency seen in postmenopausal women and is associated with an upregulation of inflammatory cytokines,^(^ ^26^ ^)^ the role of inflammation cannot be distinguished from the extensive hormonal changes. Other rodent models of inflammatory bone loss include osteoarthritis^(^ ^27^ ^)^ and colitis,^(^ ^28^, ^29^ ^)^ but the results from these models are confounded by alterations in mobility/weight‐bearing activity and nutrient malabsorption, respectively. Since chronically administered LPS induces an immune response and upregulates pro‐inflammatory cytokines with no changes in observable health status,^(^ ^30^, ^31^, ^32^, ^33^, ^34^, ^35^, ^36^ ^)^ exogenous LPS administered to rodents is a viable model for inducing systemic inflammatory bone loss to better understand the inflammatory component of osteoporosis. The objective of this systematic review and meta‐analysis was to determine whether systemic administration of LPS induced inflammatory bone loss in rodent models.

## Methods

This systematic review was conducted in accordance with PRISMA guidelines,^(^ ^37^ ^)^ and the protocol was registered in advance on PROSPERO (https://www.crd.york.ac.uk/PROSPERO/), ID CRD42020203892.

### Search strategy

The search strategy was developed with support from Brock University's library services (EF). MEDLINE, Embase, CINAHL Complete, ProQuest Dissertations and Theses Global, and Web of Science databases were searched from inception through January 13, 2021, for studies that administered exogenous LPS in vivo in rodent models and examined bone outcomes (Table 1). The full systematic search strategy across databases is presented in Table S1. Language was restricted to English. All citations returned by this search were imported into Covidence systematic review software (www.covidence.org), and duplicates were removed.

**Table 1:** Structure of Systematic Literature Search

**Lipopolysaccharide**
lipopolysaccharide OR endotoxin OR LPS OR lipoglycans OR lipid A OR O Antigen
**Bone**
bone and bones OR femur OR leg bones OR fibula OR tibia OR arm bones OR diaphysis OR epiphysis OR spine OR lumbar vertebra bone tissue OR bone density OR bone loss OR bone microarchitecture or bone histomorphometry OR cancellous bone OR spongy bone OR trabecular bone OR cortical bone OR cortical thickness OR compact bone* OR bone volume OR trabecular number OR trabecular thickness OR trabecular separation OR bone strength OR mineral apposition rate OR bone mineral OR bone structure
**Rodent Models**
rodentia OR rodent OR mouse OR mice OR mus OR rat OR rats OR guinea pig OR cricetinae OR hamster

*Note*: Search terms within each concept were linked with “OR” and concepts with “AND.” Database‐specific syntax, truncation options, and proximity operators were used.

### Eligibility criteria and study selection

All study titles and abstracts were independently screened for inclusion criteria by two independent reviewers. The principal researcher (KB) screened all studies, whereas two other researchers (SP, WW) each screened half. Any disagreements between the two reviewers on a study were resolved by the third reviewer. Studies were excluded if any of the following applied: (i) not an original study, (ii) not a full‐text publication in English, (iii) animal models other than rodents, human, or cell culture studies, (iv) no systemic in vivo delivery of exogenous LPS (local injections or diet‐induced), (v) a lethal dose of LPS causing septic shock or death, (vi) none of the specified bone outcomes reported, or (vii) no comparator group. If it was unclear from a study title and abstract whether the study should be excluded, the study was included in the full text screening. Published research articles and dissertations were considered for inclusion, whereas research in progress and conference proceedings were excluded because these forms of research had not yet undergone peer review.

Studies that passed title and abstract screening were assessed in full text by the principal reviewer (KB), and those studies meeting all inclusion criteria were then included in the synthesis. Study inclusion criteria were as follows: (i) experimental study that was performed in a rodent model (mouse, rat, guinea pig, hamster), (ii) the intervention administered exogenous LPS in vivo systemically, (iii) a control group was used for comparison, and (iv) bone outcomes were examined. Bone outcomes from the following analysis methods were included: calipers/measuring tape, dual energy X‐ray absorptiometry (DXA), micro–computed tomography (μCT), mechanical strength testing, microindentation, bone ashing, histology, dynamic bone histomorphometry, specific bone cell staining, blood biomarkers related to bone formation or resorption. In the case where a decision for study inclusion was not straightforward, input from the other reviewers (SP, WW) was sought, and decisions were made by consensus. All such decisions were documented.

### Data extraction

Data extraction was performed independently and in duplicate by two researchers (KB, RF). The following characteristics were extracted from the included studies: bibliographic information, information about the animal model (species, strain, age, sex, number of animals per group), LPS intervention (serotype, dosage, route of administration, duration, delivery schedule), and bone outcome measures (trabecular and cortical bone structure, bone quantity, bone strength, bone cell staining, serum bone biomarkers). None of the reviewed studies performed microindentation or bone ashing. Data were extracted from tables/text first, and in cases where data were only presented in a graphical format, corresponding authors were contacted via email with a request for additional information. In cases where no response was received within 2 weeks, graphical data were extracted using Web Plot Digitizer (https://apps.automeris.io/wpd/).

### Risk of bias assessment

The risk of bias of each included study was assessed using the Systematic Review Center for Laboratory animal Experimentation (SYRCLE) risk of bias tool.^(^ ^38^ ^)^ This tool consists of 10 questions to identify different types of potential bias, including selection (sequence generation, baseline characteristics, allocation concealment), performance (random housing, blinding), detection (random outcome assessment, blinding), attrition (incomplete outcome data), and reporting (selective outcome reporting), and other (other sources) bias in studies. A yes indicated a low risk of bias, no a high risk of bias, and unclear that there was insufficient information to evaluate the risk of bias. A detailed description of the types of risk of bias are summarized in Table S2.

### Statistical analysis

#### Effect estimates

For each individual study, the effect was calculated as the standardized mean difference (SMD) Hedge's *g* between the LPS and control group using Review Manager (RevMan) version 5.4 (The Cochrane Collaboration, 2020). The SMD was calculated as follows:

$$\mathrm{SE}\left\{{\mathrm{SMD}}_{i}\right\}=\sqrt{\frac{N_{i}}{n_{1i}n_{2i}}}+\frac{{\mathrm{SMD}}_{i}^{2}}{2\left(N_{i}-3.94\right)}.$$

#### Meta‐analysis

Bone outcomes were presented as mean ± standard deviation (SD). We pooled study‐specific SMD and corresponding 95% confidence intervals (CIs) with a DerSimonian and Laird random‐effects meta‐analysis for each outcome. Subgroup analysis by skeletal site was performed if there were at least three studies per skeletal site. For studies that reported multiple skeletal sites, time points, or LPS dosages, each set of data was included in the appropriate meta‐analysis, but the study was only counted once. Heterogeneity was quantified using the *I* ^2^ statistic. We pooled shorter‐term (<2 weeks) and longer‐term (>2 weeks) LPS interventions separately because of the intractable differences in design features, such as mode of administration and dose. This was supported by a meta‐analysis testing subgroup analysis by duration, which demonstrated a significant difference between studies less than 2 weeks in duration and studies greater than 2 weeks in duration for BV/TV (Fig. S1) but not vBMD (Fig. S2). Differences between these study designs are summarized in Table S3.

#### Missing data

In cases where the sample size was reported as a range, the lowest number was used. To maximize the use of data, for studies that did not report a sample size, the average sample size for studies lasting <2 weeks was assumed when missing in studies lasting <2 weeks; and the average sample size for studies lasting ≥ 2 weeks was assumed when missing in studies ≥ 2 weeks. If a study reported the sample size but no measure of variance, a pooled SD from studies that did report the SD was used:

$$\mathrm{SD}=\sqrt{\frac{\sum {\mathrm{SD}}_{i}^{2}\times \left(n_{i}-1\right)}{\left(n_{i}-n\right)}}.$$

Studies that reported standard error of the mean (SEM) were converted to SD by multiplying the SEM by the square root of the corresponding control or LPS group sample size.

#### Publication bias

Publication bias was assessed by visual inspection of funnel plots in conjunction with Egger's regression. Where publication bias was suspected, and at least 10 studies were available, we conducted a “trim‐and‐fill” analysis to account for publication bias. The method iteratively estimates the number of studies potentially missing because of publication bias at the iteration stage. Then, at the final pooling stage, the effect sizes and effect‐size standard errors are imputed (“filled in”) for these studies and an overall effect size is estimated. We used the approach of Duval and Tweedie (2000).^(^ ^39^ ^)^

#### Software

Data were analyzed using Review Manager (RevMan) version 5.4 (The Cochrane Collaboration, 2020) and Stata version 17 (StataCorp LLC, 2019).

## Results

### Study selection and characteristics

**Fig. 1:**
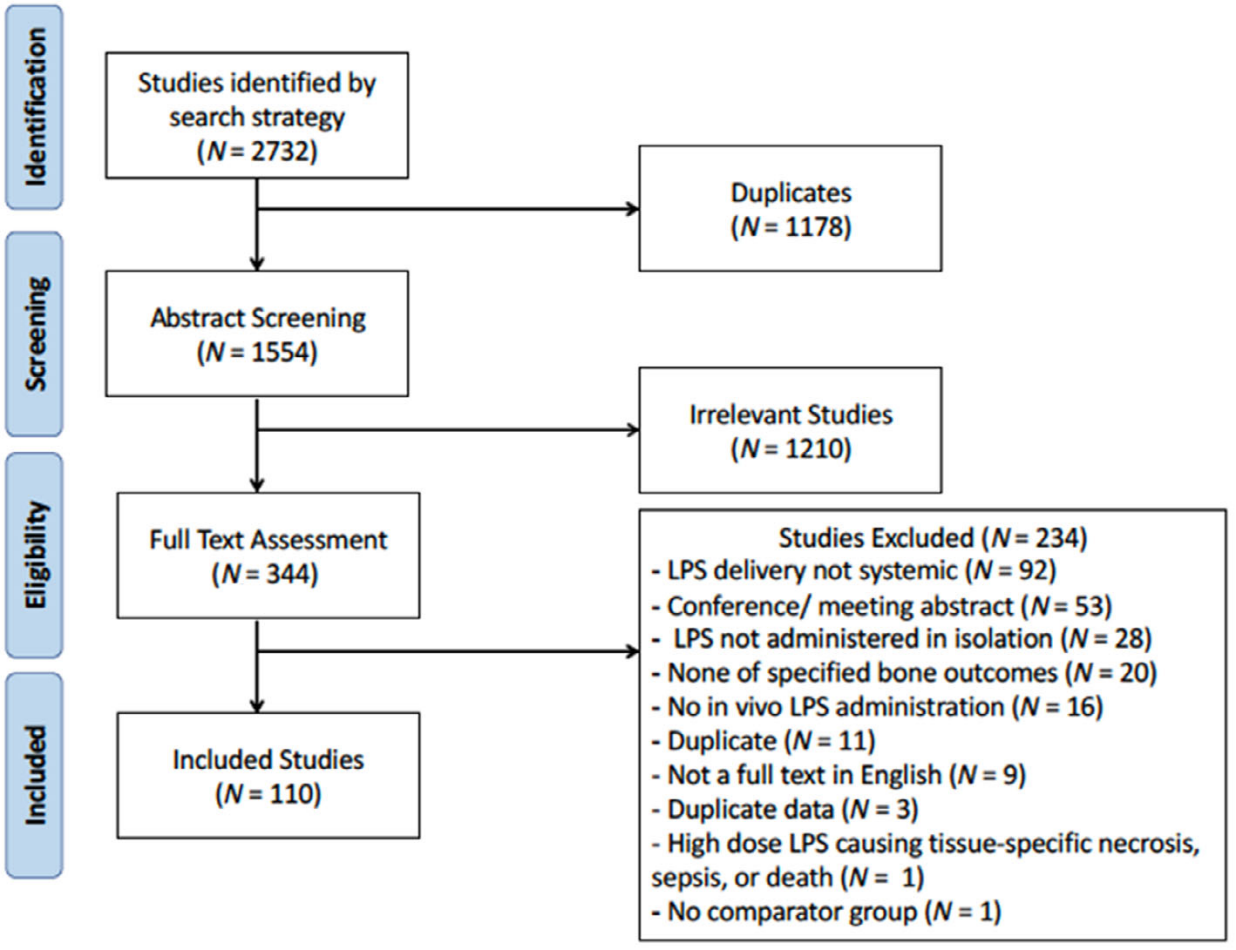
Flow chart of study screening and selection process.

The search strategy returned a total of 1554 studies for assessment after the removal of duplicates. Following title and abstract screening, 344 studies were selected for full text review, of which a total of 113 studies met the inclusion criteria. During data extraction, it was noted that there were three sets of studies that included the same control and LPS data in two papers such that we used one data set from these studies. This resulted in the exclusion of three additional studies. These studies had distinct objectives and the duplication of the control data between studies would lead to a reduction in the number of animals required. Taken together, this brought the total number to 110 studies included in the systematic review. The process of identification, screening, eligibility, and inclusion is summarized in Fig. 1.

**Fig. 2:**
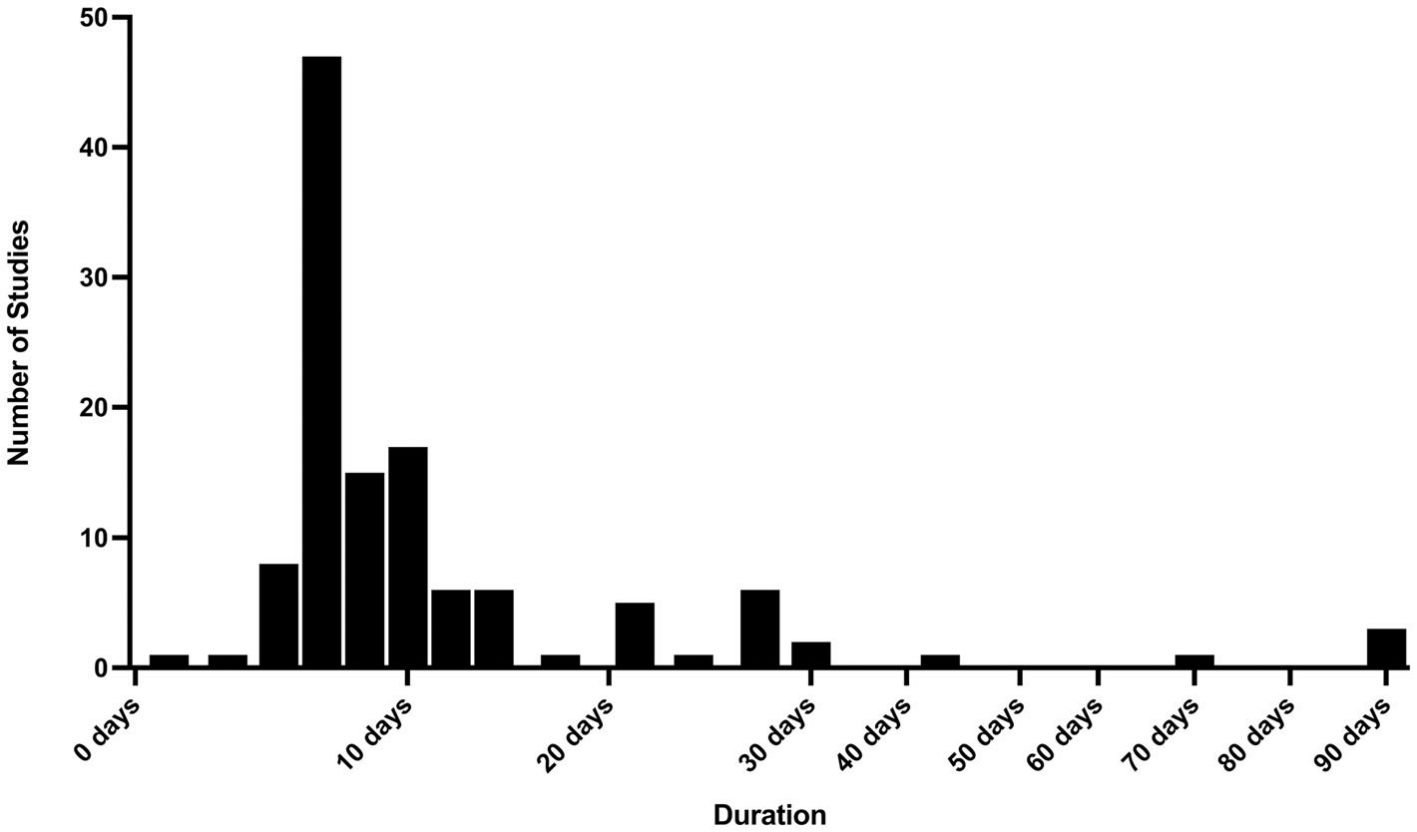
Duration of lipopolysaccharide (LPS) administration in included studies (*n* = 110). Given the clustering of studies with LPS administration less than 2 weeks with more homogeneous study design compared to studies with LPS administration greater than 2 weeks, this 2‐week cutoff was used for analysis.

Of the included studies, 83% (*n* = 91) had an experimental duration of less than 2 weeks and 17% of studies (*n* = 19) had an experimental duration of greater than 2 weeks. Two studies were included in the short‐term experiment that used a 2‐week LPS intervention but examined the effects on bone outcomes either 6 weeks^(^ ^40^ ^)^ or 8 weeks^(^ ^41^ ^)^ after the initial LPS exposure. The LPS exposure duration is summarized in Fig. 2. In terms of LPS administration, 92% of the studies (*n* = 101) used injections, whereas 8% of studies (*n* = 9) used LPS incorporated into slow‐release pellets. Only 5% of the studies (*n* = 5) were in rats, all of which were Sprague–Dawley strain, whereas 95% of the studies (*n* = 105) used mice, including ICR, C57BL/6J, BALB/c, ddY, DBA/1 J, and DBA/2 mouse strains; two studies did not specify the mouse strain. For an experimental duration of less than 2 weeks, 64% (*n* = 58) of the studies included only males, 5% (*n* = 5) of the studies included only females, and 31% (*n* = 28) of the studies did not specify the sex studied. For experimental durations of greater than 2 weeks, 19% (*n* = 6) of the studies included only males and 58% (*n* = 11) included only females, and sex was not specified in 11% (*n* = 2) of the studies.

### Risk of bias

**Fig. 3:**
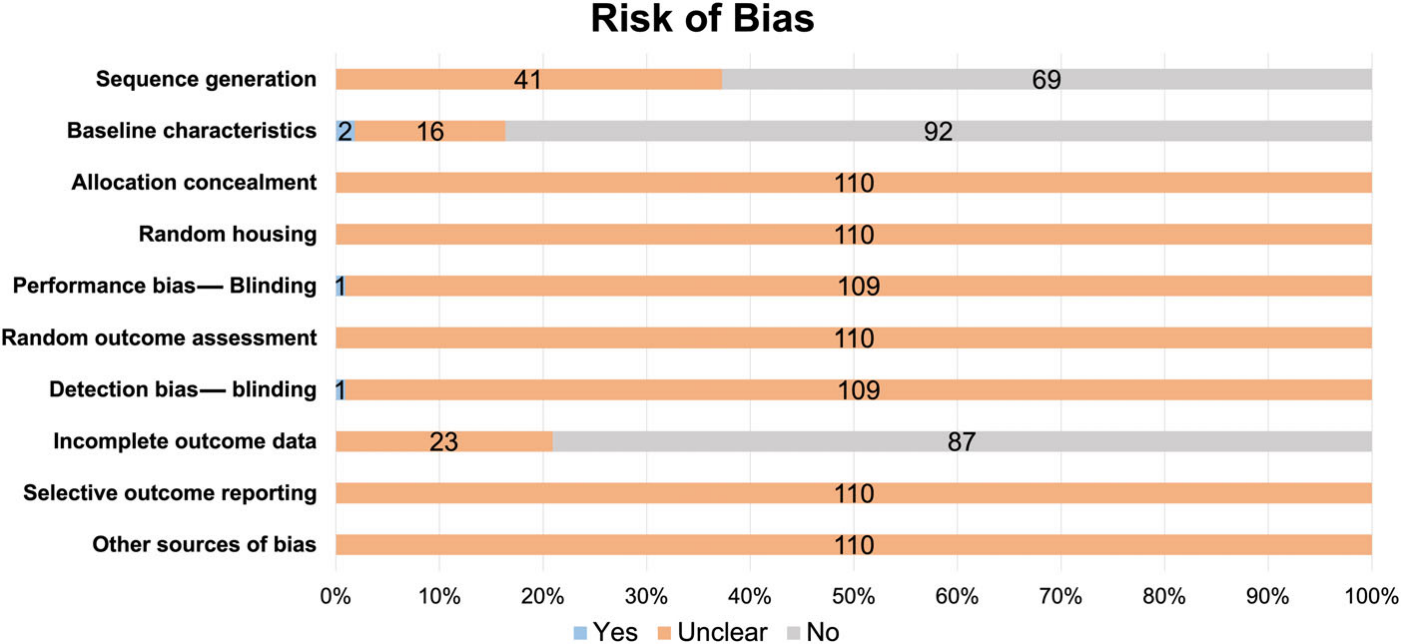
Results of the risk of bias for 110 studies according to the SYRCLE risk of bias tool. “Yes” indicates low risk of bias, “no” high risk of bias, and “unclear” an unclear risk of bias. The stacked bars represent the percentage of studies, and the numbers within each bar represent the number of studies.

All studies were considered to have an unclear risk of bias according to the SYRCLE risk of bias tool. For selection bias, 37% (*n* = 41) of the studies reported randomization to groups, but none provided information on the methods used to generate the allocation sequence. Although 17% (*n* = 19) of the studies reported body weight either at baseline or at the study endpoint, only 2% (*n* = 2) of the studies stratified randomization of animals by body weight to ensure group body weights were similar at baseline in all groups. There was no reported allocation concealment, random housing, or random outcome assessment in any of the studies. In one study, weight loss and disease severity were monitored in a “blinded manner” to avoid performance bias, and in one other study (1%), μCT analysis was blinded to avoid detection bias. Determining the attrition bias for incomplete data reporting was difficult given that only 21% (*n* = 23) of the studies reported the number of animals used in the experiment and in the data analysis. However, details were lacking, so the studies that did report the number of animals in the experimental design and analysis were classified as unclear. The reporting bias and reporting of measures against other types of bias were not mentioned in any of the studies. The results of the risk of bias evaluation are presented in Fig. 3.

### Bone structure and BMD

Skeletal sites analyzed using μCT and DXA included the tibia, femur, and lumbar vertebra. Experimental durations less than 2 weeks generally consisted of two to three injections of LPS ranging from 5 to 10 mg/kg body weight per injection with bones collected for ex vivo analysis within 2 weeks of the first LPS exposure. This type of experimental design consistently demonstrated negative effects of LPS on trabecular bone structure, including BV/TV, Tb.N, and Tb.Sp, in the femur^(^ ^42^, ^43^, ^44^, ^45^, ^46^, ^47^, ^48^, ^49^, ^50^, ^51^, ^52^, ^53^, ^54^, ^55^, ^56^, ^57^, ^58^, ^59^, ^60^, ^61^, ^62^, ^63^, ^64^, ^65^, ^66^, ^67^, ^68^, ^69^, ^70^, ^71^, ^72^, ^73^, ^74^, ^75^, ^76^, ^77^, ^78^, ^79^, ^80^, ^81^, ^82^, ^83^, ^84^, ^85^, ^86^, ^87^, ^88^, ^89^, ^90^, ^91^, ^92^, ^93^, ^94^, ^95^, ^96^, ^97^, ^98^, ^99^, ^100^, ^101^, ^102^, ^103^, ^104^, ^105^, ^106^, ^107^, ^108^, ^109^, ^110^, ^111^, ^112^, ^113^, ^114^, ^115^, ^116^, ^117^, ^118^ ^)^ and tibia.^(^ ^68^, ^119^, ^120^, ^121^, ^122^, ^123^, ^124^ ^)^ In studies that reported negative effects of LPS exposure on trabecular bone structure, approximately half (22/47) also reported a decreased Tb.Th in the femur^(^ ^48^, ^55^, ^59^, ^70^, ^77^, ^81^, ^88^, ^89^, ^94^, ^97^, ^98^, ^99^, ^100^, ^101^, ^107^, ^108^, ^114^, ^117^, ^123^ ^)^ and tibia.^(^ ^119^, ^120^, ^122^ ^)^ These alterations in trabecular bone structure were in conjunction with reduced vBMD of the femur^(^ ^48^, ^58^, ^60^, ^61^, ^65^, ^66^, ^68^, ^77^, ^79^, ^80^, ^82^, ^83^, ^95^, ^99^, ^102^, ^103^, ^106^, ^107^, ^108^, ^109^, ^114^, ^115^, ^116^, ^121^ ^)^ and tibia^(^ ^68^ ^)^ measured using μCT and areal BMD (aBMD) of the femur^(^ ^49^, ^50^, ^58^, ^111^, ^125^, ^126^ ^)^ and tibia^(^ ^124^ ^)^ measured using DXA. Cortical bone structure, including Ct.Ar/Tt.Ar and Ct.Th, remained unchanged in three out of four studies,^(^ ^98^, ^106^, ^117^ ^)^ but both studies that analyzed TMD reported negative effects of LPS exposure.^(^ ^64^, ^65^ ^)^ Notably, the two studies that utilized a LPS exposure for less than 2 weeks but examined the lingering effects either 4 or 8 weeks after the first LPS injection also reported negative effects of LPS on trabecular bone including, BV/TV, Tb.N, and Tb.Sp with no change in Tb.Th, but did not examine cortical bone.^(^ ^40^, ^41^ ^)^ It is unlikely that cortical bone would be altered within this short exposure to LPS.

Experimental durations greater than 2 weeks demonstrated negative effects of LPS on trabecular bone structure, including BV/TV, Tb.N, and Tb.Sp, in the femur,^(^ ^30^, ^32^, ^35^, ^127^, ^128^, ^129^, ^130^, ^131^ ^)^ tibia,^(^ ^33^, ^132^, ^133^ ^)^ and lumbar vertebra.^(^ ^30^, ^31^, ^132^, ^134^ ^)^ Similar to shorter‐duration LPS exposure, seven out of 11 studies that reported negative effects on trabecular bone structure also reported a decrease in Tb.Th in the femur,^(^ ^30^, ^35^, ^129^, ^130^, ^131^ ^)^ tibia,^(^ ^135^ ^)^ and lumbar vertebra.^(^ ^136^ ^)^ These alterations in bone structure were also reflected in a reduced vBMD of the femur^(^ ^127^, ^129^, ^131^, ^137^, ^138^, ^139^ ^)^ and lumbar vertebra^(^ ^134^ ^)^ measured using μCT analysis and aBMD of the femur,^(^ ^34^, ^35^, ^36^ ^)^ tibia,^(^ ^132^ ^)^ and lumbar vertebra^(^ ^31^, ^36^, ^128^, ^132^ ^)^ measured using DXA. In all eight of the studies that reported cortical bone outcomes (Ct.Ar, Tt.Ar, Ct.Ar/Tt.Ar, Ct.Th), these measures remained unaffected by LPS exposure.^(^ ^30^, ^31^, ^32^, ^35^, ^36^, ^132^, ^133^ ^)^

In particular, one study examining multiple skeletal sites reported that trabecular bone outcomes in lumbar vertebra were negatively impacted, whereas the tibia bone outcomes remained unchanged in response to the LPS exposure.^(^ ^31^ ^)^ Additionally, the effects of dose and duration in mice were demonstrated comparing LPS dosages of 0.01 mg/kg/day and 0.1 mg/kg/day for either 30 or 90 days in duration. This experimental design demonstrated a general decline in tibia trabecular bone outcomes in the 30‐day but not 90‐day LPS exposure regardless of dosage, while cortical bone outcomes were unaffected regardless of dosage or duration.^(^ ^133^ ^)^ Alternatively, in rats, 90‐day LPS exposure only demonstrated limited negative affects using a greater LPS exposure of 0.0333 mg/day but not 0.0033 mg/day.^(^ ^36^ ^)^ However, a different study in mice comparing durations of LPS exposure for 4, 6, or 10 weeks at a dosage of 0.1 mg/kg/day did not affect either trabecular or cortical bone structure or aBMD in the tibia.^(^ ^140^ ^)^ In general, LPS exposure greater than 2 weeks negatively impacted trabecular bone structure and BMD, though there are some discrepant findings among studies, and these may be attributed to the broader range in the duration, dose, and delivery of LPS in the experimental design.

### Meta‐analysis of the effects of LPS on BV/TV and vBMD

**Fig. 4:**
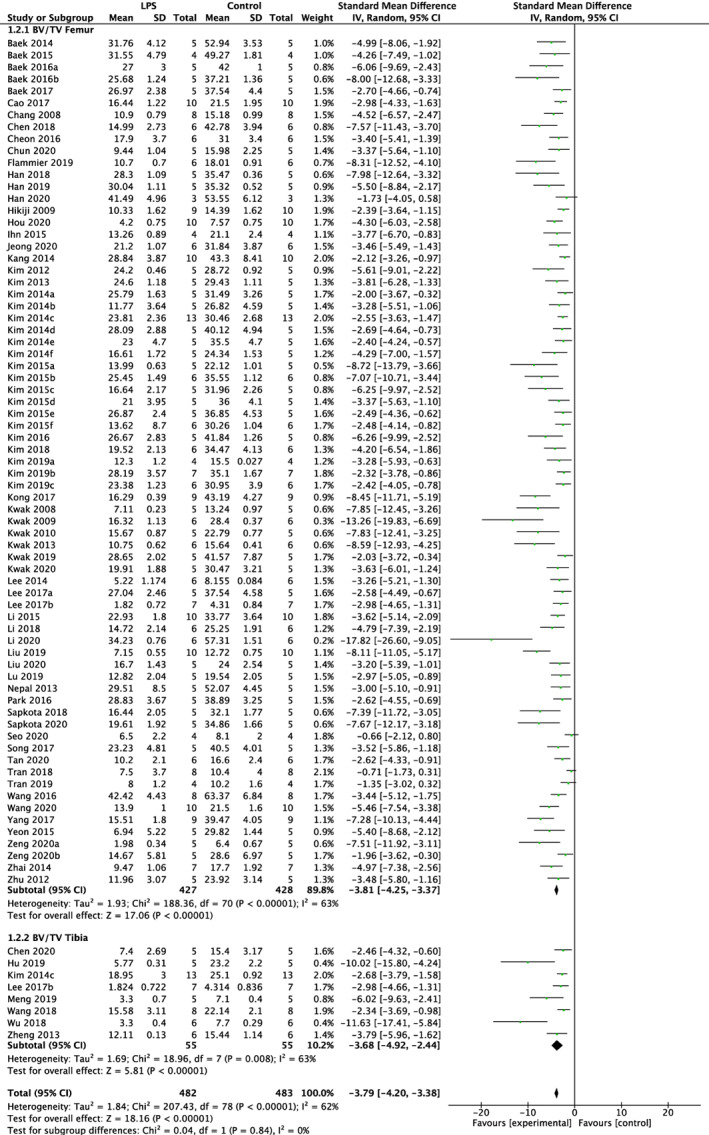
Effect of lipopolysaccharide (LPS) on BV/TV of femur and tibia in studies lasting less than 2 weeks. LPS, lipopolysaccharide; BV/TV, bone volume fraction; CI, confidence interval; SD, standard deviation; IV, weighted mean difference.

**Fig. 5:**
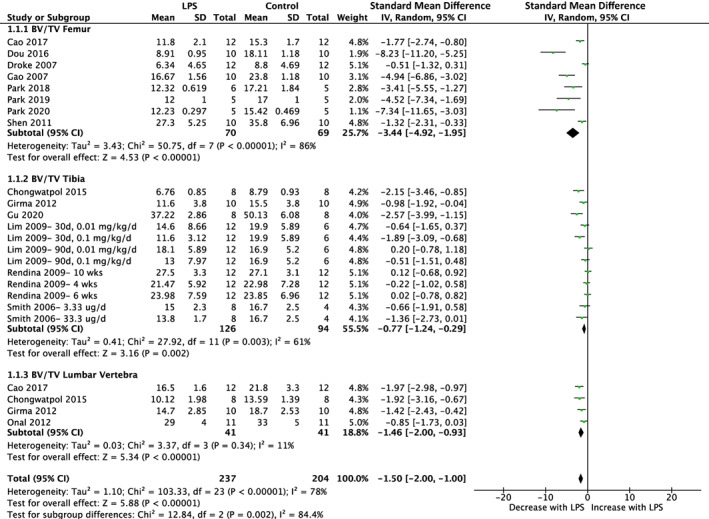
Effect of lipopolysaccharide (LPS) on vBMD of femur and tibia in studies lasting less than 2 weeks. LPS, lipopolysaccharide; vBMD, volumetric bone mineral density; CI, confidence interval; SD, standard deviation; IV, weighted mean difference.

LPS negatively impacted BV/TV regardless of experimental duration. Of the 91 studies utilizing a LPS exposure of less than 2 weeks, 79 studies reported BV/TV and 27 studies reported vBMD. The meta‐analysis indicated reduced BV/TV in the femur (SMD = −3.81%, 95% CI [−4.25, −3.37]; *p* < 0.01) with substantial heterogeneity (*I* ^2^ = 63%) and tibia (SMD = −3.68%, 95% CI [−4.92, −2.44]; *p* < 0.01) with substantial heterogeneity (*I* ^2^ = 63%). Overall LPS had a significant effect on BV/TV regardless of skeletal site (subgroup difference *p* > 0.01) in short‐term studies (SMD = −3.79%, 95% CI [−4.20, −3.38]; *p* < 0.01) with substantial heterogeneity (*I* ^2^ = 62%) (Fig. 4). Similarly, vBMD was reduced in the femur (SMD = −3.33%, 95% CI [−4.02, −2.63]; *p* < 0.01) with substantial heterogeneity (*I* ^2^ = 66%) and tibia (SMD = −1.67%, 95% CI [−3.57, 0.23]; *p* < 0.01) with substantial heterogeneity (*I* ^2^ = 87%). This resulted in a total effect of LPS regardless of skeletal site (subgroup difference *p* > 0.01) in studies less than 2 weeks in duration (SMD = −3.11%, 95% CI [−3.78, −2.44]; *p* < 0.01) with substantial heterogeneity (*I* ^2^ = 72%) (Fig. 5).

**Fig. 6:**
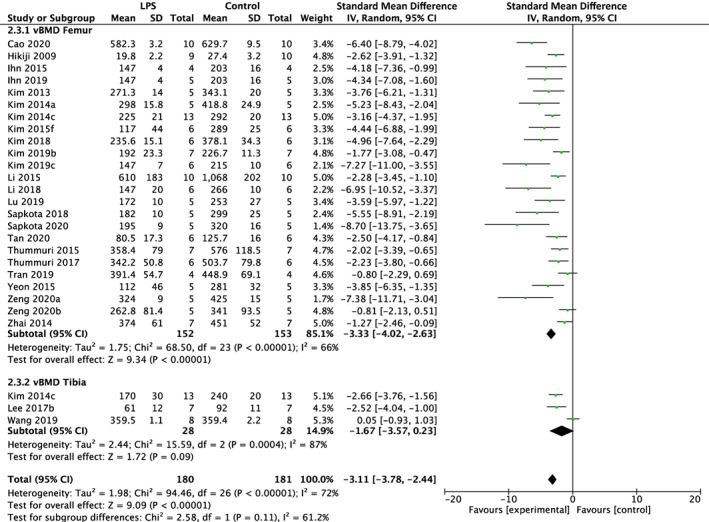
Effect of lipopolysaccharide (LPS) on BV/TV of femur, tibia, and lumbar vertebra in studies lasting more than 2 weeks. LPS, lipopolysaccharide; BV/TV, bone volume fraction; CI, confidence interval; SD, standard deviation; IV, weighted mean difference.

**Fig. 7:**
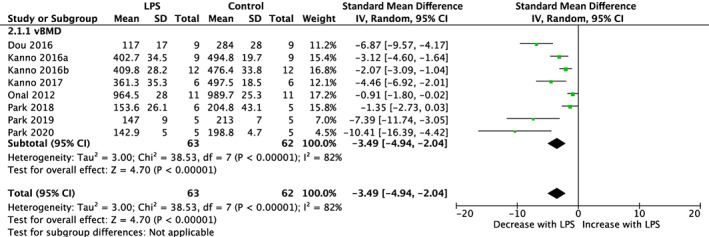
Effect of lipopolysaccharide (LPS) on vBMD of femur and lumbar vertebra in studies lasting more than 2 weeks. LPS, lipopolysaccharide; vBMD, volumetric bone mineral density; CI, confidence interval; SD, standard deviation; IV, weighted mean difference.

Of the 19 studies utilizing a LPS exposure of greater than 2 weeks, 15 reported BV/TV and eight reported vBMD. The femur demonstrated reduced BV/TV in response to LPS (SMD = −3.44%, 95% CI [−4.92, −1.95]; *p* < 0.01) with substantial heterogeneity (*I* ^2^ = 86%), as well as the tibia (SMD = −0.77%, 95% CI [−1.24, −0.29]; *p* < 0.01) with substantial heterogeneity (*I* ^2^ = 61%) and lumbar vertebra (SMD = −1.46%, 95% CI [−2.00, −0.93]; *p* < 0.01) with low heterogeneity (*I* ^2^ = 11%). Subgroup analysis for different skeletal sites was significant (*p* < 0.01) and indicated that the femur was associated with the greatest decline in BV/TV, followed by the lumbar vertebra and tibia. Overall, the effect of LPS on BV/TV in studies of longer duration resulted in reduced BV/TV (SMD = −1.50%, 95% CI [−2.00, −1.00]; *p* < 0.01) with substantial heterogeneity (*I* ^2^ = 78%) (Fig. 6). Of the studies that measured vBMD except for one study that analyzed the lumbar vertebra, all other studies analyzed the femur skeletal site. LPS reduced vBMD across skeletal sites (SMD = −3.49%, 95% CI [−4.94, −2.04]; *p* < 0.01) with substantial heterogeneity (*I* ^2^ = 82%) (Fig. 7).

According to the Egger regression results, publication bias was determined to be present in both short‐ and long‐term LPS studies for BV/TV and vBMD. Within the short‐term study duration, trim‐and‐fill analysis imputed a total of 23 studies for BV/TV while no studies were imputed for vBMD, whereas within the long‐term study duration no studies were imputed either for BV/TV or vBMD following the trim‐and‐fill analysis. Funnel plots are presented for short‐duration LPS studies (Fig. S3) and long‐duration LPS studies (Fig. S4).

### Bone histomorphometry

Studies that utilized LPS exposures for less than 2 weeks reported negative alterations in histological trabecular bone structure, including BV/TV, Tb.Th, Tb.N, and Tb.Sp, in the femur^(^ ^106^, ^126^, ^141^ ^)^ and tibia.^(^ ^119^, ^122^, ^123^, ^142^ ^)^ Notably, four of these seven studies^(^ ^106^, ^119^, ^122^, ^123^ ^)^ also reported μCT findings and demonstrated an agreement in the structure data obtained through both histomorphometry and μCT analyses. In line with these data, there was also a reduction in mineral apposition rate (MAR)^(^ ^106^ ^)^ and an increase in eroded surface/bone surface (ES/BS),^(^ ^65^, ^67^, ^70^, ^81^, ^86^, ^88^, ^94^, ^96^, ^100^, ^102^, ^103^, ^118^ ^)^ along with an increase in both the number of osteoclasts^(^ ^41^, ^42^, ^43^, ^44^, ^45^, ^46^, ^52^, ^56^, ^57^, ^60^, ^61^, ^62^, ^63^, ^69^, ^70^, ^75^, ^76^, ^77^, ^78^, ^86^, ^88^, ^89^, ^90^, ^93^, ^101^, ^102^, ^103^, ^104^, ^105^, ^106^, ^111^, ^113^, ^117^, ^121^, ^122^, ^123^, ^126^, ^141^, ^142^ ^)^ and osteoclast surface.^(^ ^50^, ^54^, ^65^, ^67^, ^81^, ^87^, ^106^, ^107^, ^117^, ^118^, ^122^, ^123^, ^142^ ^)^ Only one study reported no change in osteoblast surface.^(^ ^118^ ^)^

One study utilizing a LPS exposure of greater than 2 weeks measured histological trabecular bone structure in the tibia. There was a negative effect of LPS (0.0333 mg/day) over a duration of 12 weeks on trabecular BV/TV, Tb.Th, and Tb.Sp, with no alterations in cortical bone parameters. Within this same study, these histological data support the femur μCT analysis that reported negative alterations in femur trabecular structure and a lack of change in cortical bone.^(^ ^35^ ^)^ Dynamic bone parameters were also analyzed in the tibia, with no change in the periosteal cortical dynamic bone histomorphometry measures, including MAR, mineralized surface/bone surface MS/BS, and bone formation rate/bone surface and an increase in endocortical MAR, MS/BS, and ES/BS in response to LPS exposure,^(^ ^35^ ^)^ whereas other studies reported an increased ES/BS,^(^ ^35^ ^)^ as well as the number of osteoclasts^(^ ^129^, ^131^, ^139^, ^143^ ^)^ and osteoclast surfaces.^(^ ^127^, ^129^, ^143^ ^)^ None of the longer‐term LPS exposure studies reported osteoblast bone cell staining.

### Bone strength

Few studies measured bone strength, with only one study testing the mechanical strength and other studies using finite‐element analysis to simulate the mechanical strength of a skeletal site. A study by Wang et al.^(^ ^40^ ^)^ found that total force prior to fracture, but not stiffness or elastic modulus, was reduced following LPS exposure. The duration of LPS exposure was less than 2 weeks and femur bone strength were measured 6 weeks after the initial dose of LPS.

Studies that had a LPS exposure of greater than 2 weeks and assessed bone strength used finite‐element analysis as a surrogate measure of mechanical strength. Studies that reported negative alterations in trabecular bone structure also consistently reported reduced total force prior to fracture, physiological force, and/or size‐independent stiffness in the femur,^(^ ^32^, ^35^ ^)^ tibia,^(^ ^31^ ^)^ and lumbar vertebra.^(^ ^31^, ^132^ ^)^ Stiffness was reduced in three out of four studies in the femur,^(^ ^32^ ^)^ tibia,^(^ ^31^, ^36^ ^)^ and lumbar vertebra,^(^ ^31^ ^)^ whereas von Mises stress was increased in three out of four studies that reported this measure in the femur,^(^ ^32^ ^)^ tibia,^(^ ^36^ ^)^ and lumbar vertebra.^(^ ^31^ ^)^ Interestingly, in two studies that reported nonsignificant changes in tibia trabecular bone structure, there was reduced total force, physiological force, stiffness, and size‐independent stiffness and increased von Mises stresses in response to LPS.^(^ ^31^, ^36^ ^)^ In contrast, Rendina et al.^(^ ^144^ ^)^ reported no changes in any measures of bone strength (total force and size independent stiffness), and these findings aligned with the findings that trabecular bone structure was also unchanged with LPS exposure.

### Serum biomarkers

Negative alterations in bone structure and strength associated with short‐term LPS exposure for less than 2 weeks were supported by an increase in bone resorption markers and an overall decrease in bone formation markers. Serum biomarkers for bone resorption, including receptor activator of nuclear factor kappa B ligand (RANKL)^(^ ^41^, ^43^, ^73^, ^76^, ^84^, ^98^, ^105^ ^)^ and cross‐linked c‐telopeptide of type I collagen (CTX‐1),^(^ ^57^, ^76^, ^77^, ^88^, ^105^ ^)^ were elevated, whereas osteoprotegerin (OPG),^(^ ^43^, ^76^, ^84^, ^98^, ^105^ ^)^ a serum biomarker for the inhibition of bone resorption, was reduced in five out of six studies in response to LPS exposure. Overall, there was an increase in the RANKL to OPG ratio favoring bone resorption over bone formation.^(^ ^43^, ^73^, ^76^, ^84^, ^98^, ^105^ ^)^

The effects of LPS exposure for more than 2 weeks resulted in an increase in serum markers of bone resorption and no change in serum markers of bone formation. Specifically, serum CTX‐1 was elevated in all studies,^(^ ^128^, ^129^, ^130^, ^131^ ^)^ whereas one out of two studies reported an elevation in serum tartrate‐resistant acid phosphatase (TRAP).^(^ ^34^ ^)^ In terms of serum bone formation markers, bone specific alkaline phosphatase^(^ ^30^ ^)^ and alkaline phosphatase^(^ ^129^, ^130^, ^131^ ^)^ were unchanged in all studies, whereas OC remained unchanged in response to LPS in four out of five studies.^(^ ^128^, ^129^, ^130^, ^131^ ^)^

## Discussion

Rodent models are integral to current research investigating the underlying inflammatory component of osteoporosis. Our systematic review found that regardless of study duration, LPS negatively impacted trabecular bone structure and BMD and upregulated bone resorption. The negative alterations in bone outcomes reported in the rodent models mimic the characteristics of osteoporosis in humans, including reduced BMD with weakened trabecular bone structure, specifically reduced Tb.N and Tb.Th. In humans, there is greater bone loss with aging due to a shift in the ratio of bone resorption to bone formation.^(^ ^145^ ^)^

The negative impact of LPS on bone outcomes was more consistent and pronounced among studies that lasted less than 2 weeks. This may be explained by the relative homogeneity of the designs and LPS interventions among these studies, which generally consisted of two to three injections utilizing a LPS dosage ranging from 5 to 10 mg/kg body, resulting in a higher LPS exposure. These short‐term studies often used in vivo LPS exposure as a proof of concept to support cell culture work. However, osteoporosis is a chronic disease, and the studies that lasted more than 2 weeks may better represent the pathophysiology of this disease.

Studies greater than 2 weeks in duration had a more heterogeneous LPS intervention design, including delivery (injections or slow‐release pellets), LPS dosage, and study duration (ranging from 16 days to 13 weeks). LPS exposure could not be compared across studies since some dosages were reported as an absolute LPS exposure (e.g., mg/day) versus a relative LPS exposure (e.g., mg/kg body weight/day). Although the relative exposure accounts for body size, slow‐release pellets implanted at the beginning of the study would not be adjustable for changes in body weight over the course of the study. A benefit of the slow‐release pellets is that they offer long‐term LPS exposure compared to injections. However, research testing the delivery rate of 17β‐estradiol via slow‐release pellets reported an initial bolus release followed by a substantial decline.^(^ ^146^, ^147^ ^)^

Differences in study duration and design were supported by the meta‐analysis of BV/TV, which indicated that the short‐term studies that were less than 2 weeks in duration had a greater effect size based on the SMD and 95% CI compared to the longer‐term studies that lasted more than 2 weeks. Of the trabecular bone outcomes only BV/TV was included in the meta‐analysis since the other structural outcomes (Tb.N, Tb.Th, Tb.Sp) influence BV/TV. In addition, although osteoporosis in humans is assessed using aBMD quantified by DXA, this measure is not typically reported in rodent studies because it is not sensitive enough to capture changes in bone. Additionally, there were only enough studies to conduct a meta‐analysis on vBMD, not aBMD. We found no difference in skeletal site subgroup analysis for the short‐term studies, but the long‐term studies indicated a greater effect of LPS in the femur compared to the lumbar vertebra and tibia. However, it is important to consider both the substantial heterogeneity among studies when interpreting these results and the small sample size for some of the subgroup analyses that may influence the *I* ^2^ statistic. This encompasses multiple study design aspects, including differences in LPS delivery method (injection versus slow‐release pellet), LPS dose, LPS delivery schedule (multiple injections versus continuous exposure), and animal characteristics such as species (mouse versus rat), strain (inbred versus outbred), age, and sex (summarized in Table S3). In particular, animal strain can influence the inflammatory effect of LPS, given that some inbred strains, such as the C57BL/6J mouse, have blunted inflammatory responses,^(^ ^148^ ^)^ although none of the studies analyzed in this systematic review utilized the C3H/HeJ or C57BL/10ScCr mouse strains because these models are known to carry TLR4 mutations, making them resistant to LPS.^(^ ^149^ ^)^

The effects of study duration are difficult to assess across studies because of several additional important design features that may not be a consequence of duration (e.g., LPS dosing regimen and/or administration method). Conversely, Lim et al.^(^ ^133^ ^)^ compared both duration (30 days and 90 days) and LPS dosage (0.01 and 0.1 mg/kg/day slow‐release pellets) and reported a general decline in tibia trabecular bone outcomes. In contrast, Rendina et al.^(^ ^140^ ^)^ used the same dosage (0.1 mg/kg/day) as the aforementioned study, examining LPS durations of 4, 6, or 10 weeks; however, there was no effect on bone outcomes. Although both studies used C57BL/6J mice that were similar in age, Lim et al.^(^ ^133^ ^)^ used male mice while Rendina et al.^(^ ^140^ ^)^ used hormonally intact female mice. The protective anti‐inflammatory role of estrogen signaling may in part explain the discrepancy between the two studies,^(^ ^82^ ^)^ highlighting the need to consider sex when designing experiments.

Although we chose to focus on systemic LPS exposure and did not include studies using localized LPS injections (e.g., direct injections in the gingiva to induce periodontitis), skeletal site may also be differentially affected by LPS. This may be partially due to differences in weight bearing and loading patterns of different skeletal sites.^(^ ^150^ ^)^ Chongwatpol et al.^(^ ^31^ ^)^ reported that the lumbar vertebra but not the tibia was negatively impacted by LPS exposure. Since the lumbar vertebra is a less‐weight‐bearing site compared to the tibia, the lumbar vertebra would have less mechanical loading, which promotes bone formation and may in part explain the detrimental effect of the LPS exposure measured at this site and not the tibia.

Bone strength is a function of both bone quantity (mineral) and bone quality (three‐dimensional structure).^(^ ^151^ ^)^ Most of the included studies in this systematic review analyzed bone quantity and quality, but few assessed the mechanical properties of bone that provide helpful insight into the actual strength at a skeletal site. Compression testing would provide useful data about trabecular strength, while a three‐ or four‐point bending test would measure cortical strength. Of the reported studies in this systematic review, only one reported mechanical strength testing using a three‐point bending test,^(^ ^40^ ^)^ while three studies simulated trabecular compression testing using finite‐element analysis.^(^ ^31^, ^32^, ^132^ ^)^ However, previous work examining human osteoporotic specimens with femoral neck fractures demonstrated that cancellous bone microarchitecture, including BV/TV, Tb.N, and Tb.Sp, were correlated with fracture toughness (i.e., the ability to resist fracture following a crack).^(^ ^152^ ^)^ This relationship between bone structure and mechanical properties suggests that the bone structure analysis is translatable to bone strength. Reductions in bone strength, compromised bone structure, and lower BMD with both short‐ and longer‐term LPS exposure was supported by an increase in bone resorption as demonstrated by a consistent increase in serum bone resorption markers, histological osteoclast staining, and eroded bone surface in all studies that assessed these outcomes.

The increase in bone resorption was demonstrated by the upregulation in osteoclast activity through serum biomarkers and bone‐specific staining, while osteoblast activity remained relatively unchanged through serum biomarkers. The increase in RANKL to OPG ratio favored bone resorption, and osteoclast staining in bone was also increased. Previous cell culture studies demonstrated the potent effect of LPS upregulating osteoclasts,^(^ ^20^ ^)^ which is in line with these results across studies. However, exogenous LPS exposure did not inhibit osteoblasts as previously described using cell culture.^(^ ^21^ ^)^

In humans, aging is associated with an elevated circulating LPS concentration, which is suggested to contribute to the pro‐inflammatory state associated with aging.^(^ ^18^ ^)^ Additionally, an increase in gut permeability has also been proposed to contribute to age‐ related inflammation.^(^ ^19^ ^)^ LPS is a component of gram‐negative bacteria located in the gut,^(^ ^17^ ^)^ and with aging there is an increase in LPS translocation from the intestines into circulation.^(^ ^18^ ^)^ Since the prevalence of osteoporosis increases with age and emerging evidence supports the role of inflammation in the pathophysiology of this disease, developing a rodent model for LPS‐induced bone loss is pertinent to understanding osteoporosis. Furthermore, it will be important to consider the role of both inflammation and estrogen deficiency in future models to elucidate the relationship between these two aspects related to osteoporosis. The selection of a rodent model is an important consideration given that estrogen deficiency differentially affects some species and strains; in particular, female C57BL/6J mice lose bone at a young age and develop spontaneous osteopenia.^(^ ^153^ ^)^

Even though there was no time restriction, included studies were all published after 2003, with the majority being published in 2020. This may in part be explained by a shift toward understanding the underlying inflammatory pathophysiology of osteoporosis and increased accessibility to μCT and DXA analysis. Of note, none of the studies analyzed bone outcomes in vivo to measure changes longitudinally. Although at the time of this systematic search a study from our lab longitudinally measuring in vivo bone outcomes in response to exogenous LPS delivered via osmotic pumps had not yet been published, we found that trabecular and cortical bone outcomes were unaffected in male and female CD‐1 mice.^(^ ^154^ ^)^ As previously stated, slow‐release pellets are suggested to have a less consistent release rate than other delivery methods, and we hypothesized that, given the more consistent release rate of osmotic pumps, a higher dosage of LPS would be required to induce alterations in bone outcomes. This should be a future consideration in study design to better understand the development of bone loss in response to LPS and inflammation.

### Strengths and limitations

The comprehensive set of bone outcomes analyzed was a major strength of this systematic review. The encompassing set of analysis techniques allowed for both the physical (structure and BMD) and physiological (bone‐specific staining, serum biomarkers) aspects of bone to be assessed.

There are several limitations of this systematic review and meta‐analysis. In general, the sample size of the included studies was small, and in some studies the sample size was not reported. In terms of study design, it was most often the LPS bacterial strain and not the serotype that was reported, and in some cases the LPS dosage and duration were unclear, whereas for animal characteristics the sex of the animals was sometimes not specified, and body weight was not usually reported. In terms of bone outcome analysis, some studies did not report the μCT scanning parameters, and in some cases no quantitative data were reported and only μCT images of the bone were published. Additionally, only the BV/TV outcome met the criteria for meta‐analysis for both short‐term study durations of less than 2 weeks and longer study durations of more than 2 weeks, and substantial heterogeneity was reported. Overall, there was a substantial unclear risk of bias identified using the SYRCLE risk of bias tool.^(^ ^38^ ^)^ The missing information is pivotal for interpreting the results, comparing different studies, and reproducibility. This highlights the need for more rigorous reporting and study design considerations in preclinical models to reduce bias and strengthen findings from future systematic reviews. Future studies should follow the Animal Research: Reporting of In Vivo Experiments (ARRIVE) guidelines^(^ ^155^ ^)^ to ensure study reproducibility and improved research reporting. Finally, language may have been a limitation as the systematic review was restricted to English, so studies meeting the search criteria that were not in English were excluded.

## Conclusions

This systematic review found exogenous LPS administration in rodents to be a viable model for studying inflammatory bone loss to better understand the inflammatory pathophysiology of osteoporosis. We found that LPS exposure regardless of duration negatively impacted bone outcomes, including trabecular bone structure at multiple skeletal sites and BMD, and upregulated osteoclast activity, as seen in specific bone cell staining and serum biomarkers.

## Author Contributions

**Evelyn Feldman:** Methodology; resources; writing – review and editing. **Russel J. De Souza:** Formal analysis; methodology; writing – review and editing. **Elena M. Comelli:** Supervision; writing – review and editing. **Panagiota Klentrou:** Supervision; writing – review and editing. **Sandra J Peters:** Conceptualization; data curation; funding acquisition; supervision; writing – review and editing. **Wendy E Ward:** Conceptualization; funding acquisition; supervision; writing – review and editing.

## Conflicts of Interest

E.M.C. received research support from Lallemand Health Solutions and Ocean Spray and consultant fees or speaker and travel support from Danone and Lallemand Health Solutions. (All are outside of this study.)

R.J.d.S. has served as an external resource person to the World Health Organization's (WHO) Nutrition Guidelines Advisory Group on *trans* fats, saturated fats, and polyunsaturated fats. The WHO paid for his travel and accommodations to attend meetings from 2012–2017 to present and discuss this work. He presented updates of this work to the WHO in 2022. He has also done contract research for the Canadian Institutes of Health Research's Institute of Nutrition, Metabolism, and Diabetes, Health Canada, and the WHO, for which he received remuneration. He has received speaker's fees from the University of Toronto and McMaster Children's Hospital. He has held grants from the Canadian Institutes of Health Research, Canadian Foundation for Dietetic Research, Population Health Research Institute, and Hamilton Health Sciences Corporation as a principal investigator and is a co‐investigator on several funded team grants from the Canadian Institutes of Health Research. He has served as an independent director of the Helderleigh Foundation (Canada). He serves as a member of the Nutrition Science Advisory Committee to Health Canada (Government of Canada) and a co‐opted member of the Scientific Advisory Committee on Nutrition (SACN) Subgroup on the Framework for the Evaluation of Evidence (Public Health England). (All are outside of this study.)

## Author Roles

K.N.B., conceptualization, project administration, data curation, formal analysis, investigation, methodology, visualization, writing—original draft.

E.F., methodology, resources, writing—review and editing.

R.J.d.S., methodology, formal analysis, writing—review and editing.

E.M.C., supervision, writing—review and editing.

N.P., supervision, writing—review and editing.

S.J.P., conceptualization, financial acquisition, data curation, supervision, writing—review and editing.

W.E.W., conceptualization, financial acquisition, data curation, supervision, writing—review and editing.

### Peer Review

The peer review history for this article is available at https://publons.com/publon/10.1002/jbmr.4740.

## Supporting information


**Figure S1.** Subgroup analysis by duration of lipopolysaccharide (LPS) for studies less than 2 weeks and greater than 2 weeks in duration on BV/TV. LPS, lipopolysaccharide; BV/TV, bone volume fraction; CI, confidence interval; SD, standard deviation; IV, weighted mean difference.

**Figure S2.** Subgroup analysis by duration of lipopolysaccharide (LPS) for studies less than 2 weeks and greater than 2 weeks in duration on vBMD. LPS, lipopolysaccharide; vBMD, volumetric bone mineral density; CI confidence interval; SD, standard deviation; IV, weighted mean difference.

**Figure S3.** Funnel plots for studies shorter than 2 weeks in duration. Contour‐enhanced funnel plot for studies for (*A*) BV/TV and (*B*) vBMD. (*C*) Trim and fill for BV/TV (23 studies imputed; observed SMD = −4.106, 95% CI [−4.607, −3.605]; imputed SMD = −2.979, 95% CI [−3.662, −2.295]) and (*D*) vBMD (no missing studies imputed). LPS, lipopolysaccharide; BV/TV, bone volume fraction; SMD, standardized mean difference, calculated as Hedge's *g*; CI confidence interval.

**Figure S4.** Funnel plots for studies greater than 2 weeks in duration. Contour‐enhanced funnel plot for (*A*) BV/TV and (*B*) vBMD. (*C*) Trim and fill for BV/TV and (*D*) vBMD. LPS, lipopolysaccharide; vBMD, volumetric bone mineral density (no missing studies were imputed for BV/TV or vBMD); SE, standard error; SMD, standardized mean difference, calculated as Hedge's *g*.

**Table S1.** Full systematic search strategy by database.

**Table S2.** Summary of risk of bias assessment guidelines.

**Table S3.** Systematic review summary of study characteristics. Sample size represents number of studies.

## Data Availability

The data that support the findings of this study are openly available in Brock University Dataverse at https://doi.org/10.5683/SP3/REEPYJ.
