# Supplementary figures and images for: Lipopolysaccharide‐Induced Bone Loss in Rodent Models: A Systematic Review and Meta‐Analysis
Source: J Bone Miner Res. 2022 Dec 5;38(1):198–213. doi: 10.1002/jbmr.4740 (PMC10107812; doi:10.1002/jbmr.4740)

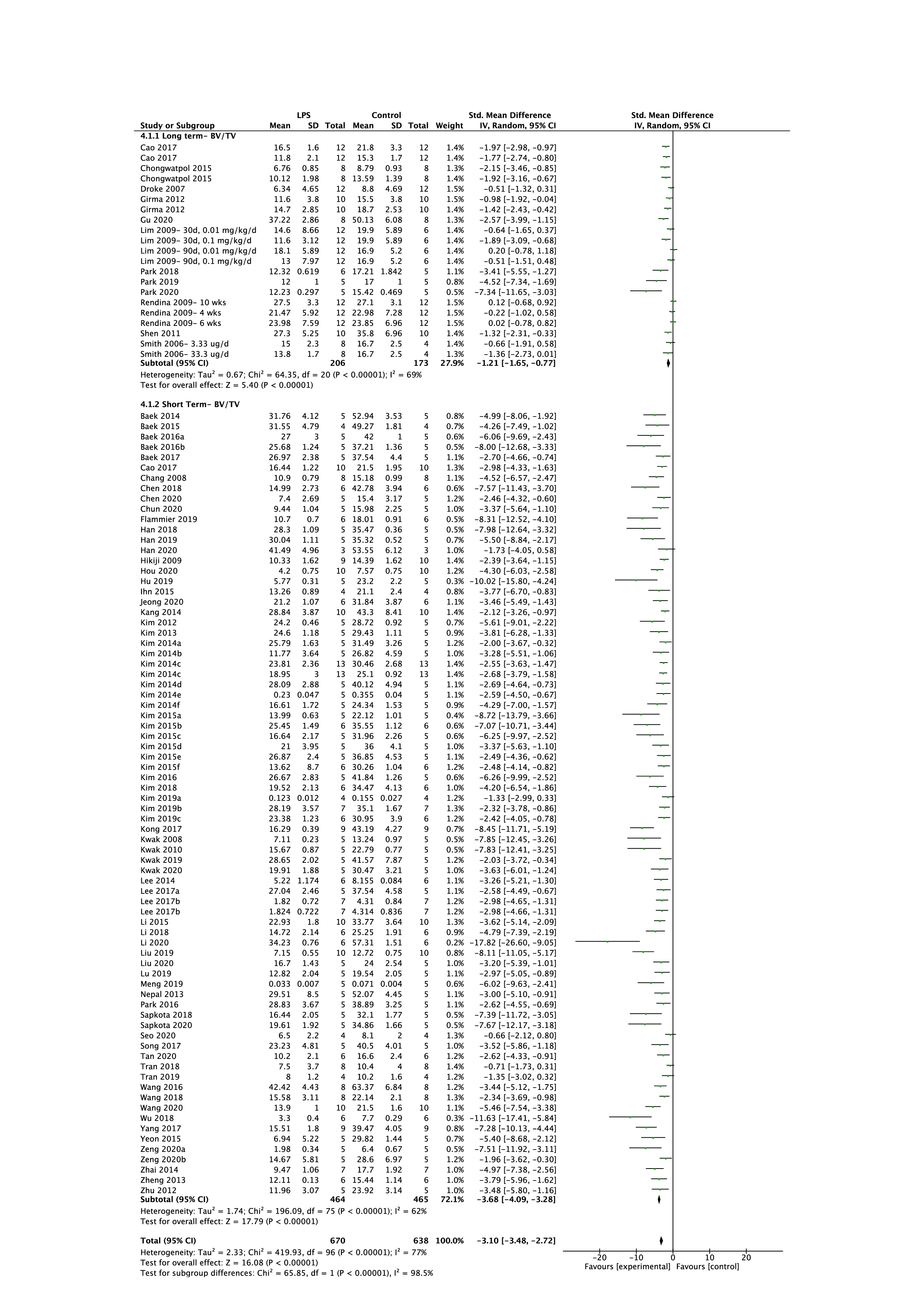

Supplement: Supplementary file 1 — Figure S1. Subgroup analysis by duration of lipopolysaccharide (LPS) for studies less than 2 weeks and greater than 2 weeks in duration on BV/TV. LPS, lipopolysaccharide; BV/TV, bone volume fraction; CI, confidence interval; SD, standard deviation; IV, weighted mean difference. [file JBMR-38-198-s004.tiff]

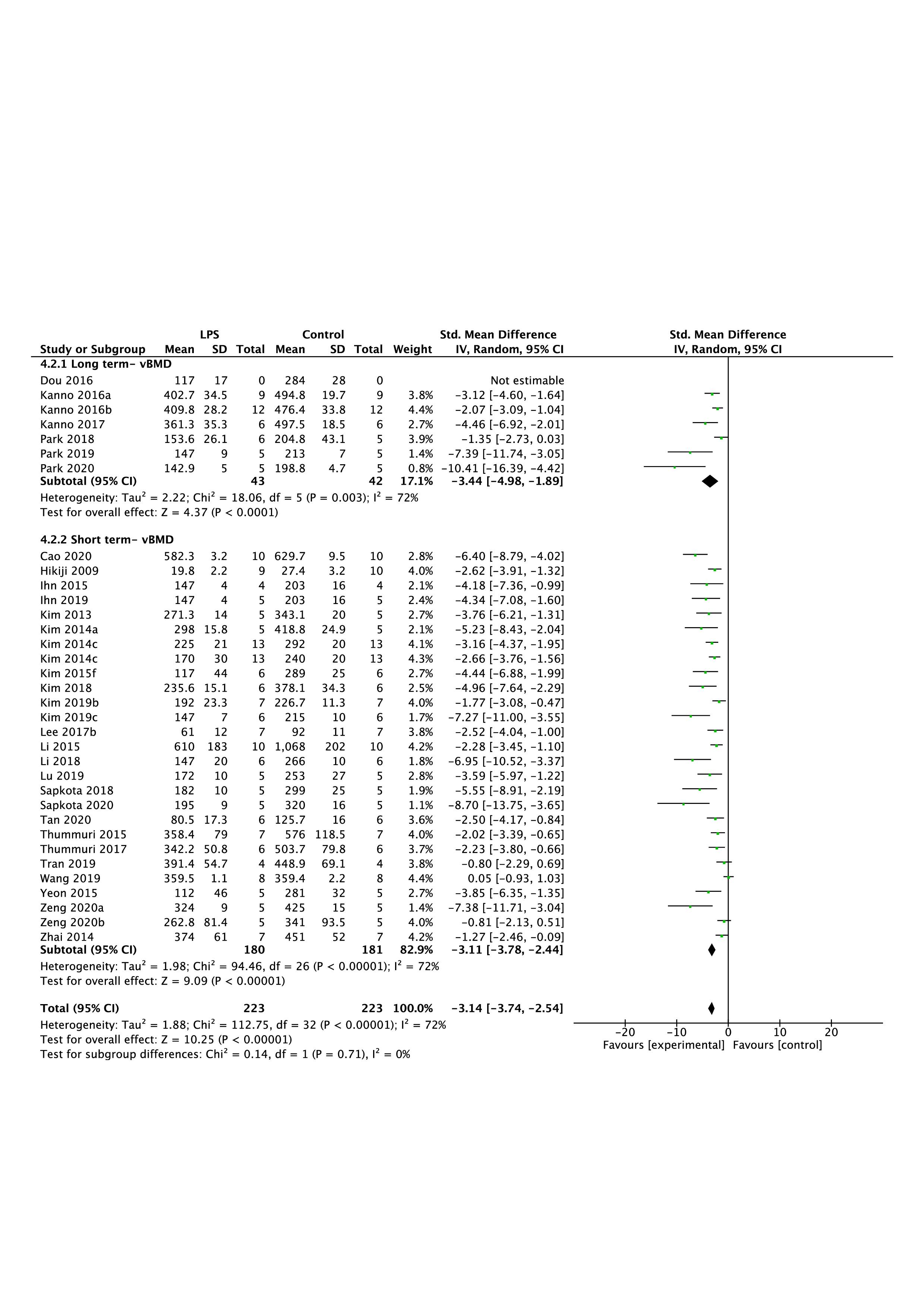

Supplement: Supplementary file 2 — Figure S2. Subgroup analysis by duration of lipopolysaccharide (LPS) for studies less than 2 weeks and greater than 2 weeks in duration on vBMD. LPS, lipopolysaccharide; vBMD, volumetric bone mineral density; CI confidence interval; SD, standard deviation; IV, weighted mean difference. [file JBMR-38-198-s007.tiff]
