# Supplementary material for: Lipopolysaccharide‐Induced Bone Loss in Rodent Models: A Systematic Review and Meta‐Analysis
Source: J Bone Miner Res. 2022 Dec 5;38(1):198–213. doi: 10.1002/jbmr.4740 (PMC10107812; doi:10.1002/jbmr.4740)
Supplement: Supplementary file 3 — Figure S3. Funnel plots for studies shorter than 2 weeks in duration. Contour‐enhanced funnel plot for studies for (A) BV/TV and (B) vBMD. (C) Trim and fill for BV/TV (23 studies imputed; observed SMD = −4.106, 95% CI [−4.607, −3.605]; imputed SMD = −2.979, 95% CI [−3.662, −2.295]) and (D) vBMD (no missing studies imputed). LPS, lipopolysaccharide; BV/TV, bone volume fraction; SMD, standardized mean difference, calculated as Hedge's g; CI confidence interval. [file JBMR-38-198-s006.docx]

**
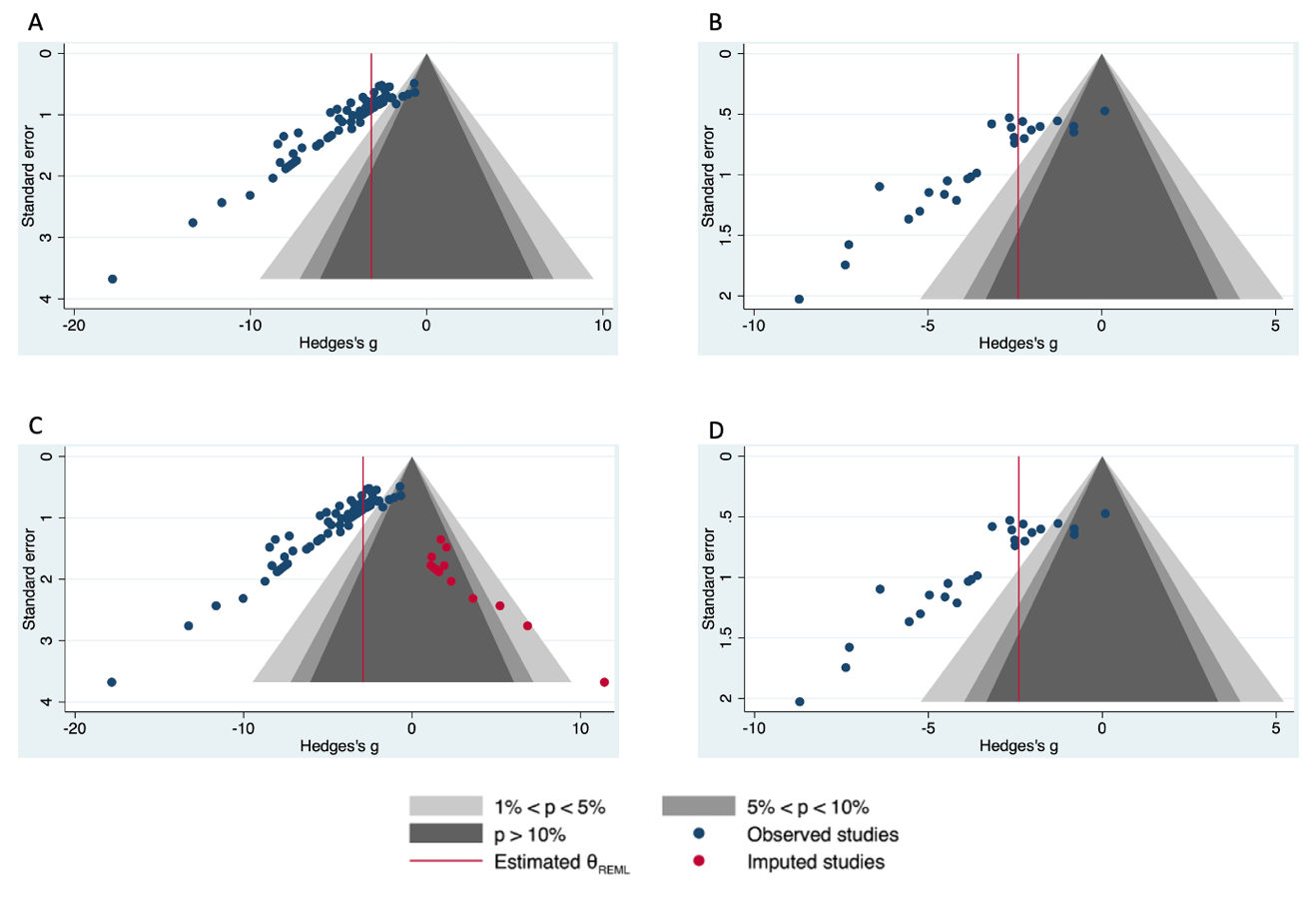
**

**Supplementary Figure 3. Funnel plots for studies shorter than 2 weeks in duration. (A)** Contour enhanced funnel plot for studies for BV/TV and **(B)** vBMD. **(C)** Trim and fill for BV/TV (23 studies imputed; Observed SMD = -4.106, 95% CI [-4.607, -3.605]; Imputed SMD = -2.979, 95% CI [-3.662, -2.295] and **(D)** vBMD (no missing studies imputed). LPS (lipopolysaccharide), BV/TV (bone volume fraction), SMD (standardized mean difference) calculated as Hedge’s *g*, CI (confidence interval).
