# Supplementary material for: Lipopolysaccharide‐Induced Bone Loss in Rodent Models: A Systematic Review and Meta‐Analysis
Source: J Bone Miner Res. 2022 Dec 5;38(1):198–213. doi: 10.1002/jbmr.4740 (PMC10107812; doi:10.1002/jbmr.4740)
Supplement: Supplementary file 4 — Figure S4. Funnel plots for studies greater than 2 weeks in duration. Contour‐enhanced funnel plot for (A) BV/TV and (B) vBMD. (C) Trim and fill for BV/TV and (D) vBMD. LPS, lipopolysaccharide; vBMD, volumetric bone mineral density (no missing studies were imputed for BV/TV or vBMD); SE, standard error; SMD, standardized mean difference, calculated as Hedge's g. [file JBMR-38-198-s002.docx]

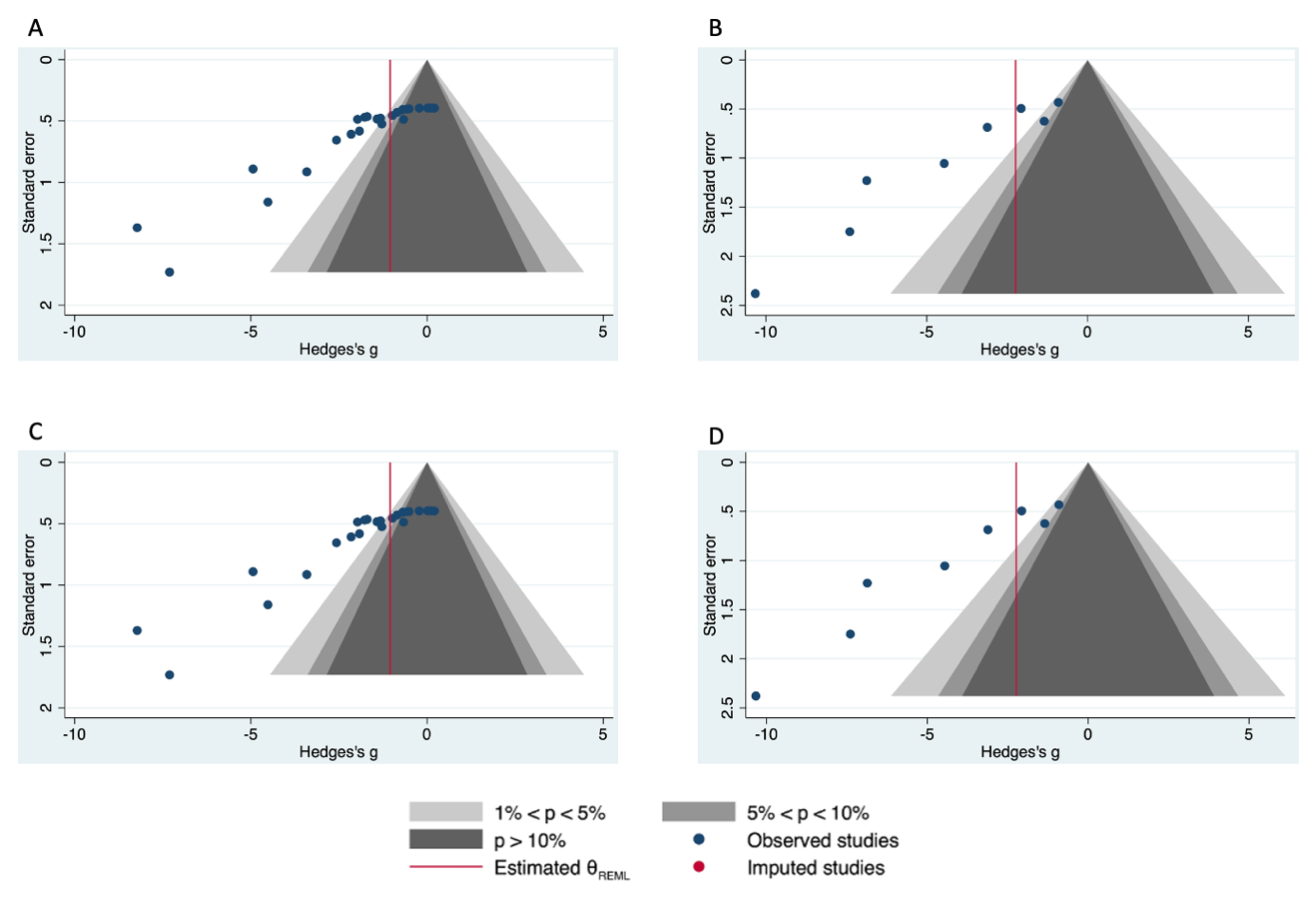


**Supplementary Figure 4. Funnel plots for studies greater than 2 weeks in duration. (A)** Contour enhanced funnel plot for BV/TV and **(B)** vBMD. **(C)** Trim and fill for BV/TV and **(D)** vBMD**.** LPS (lipopolysaccharide), vBMD (volumetric bone mineral density). No missing studies were imputed for BV/TV or vBMD. SE (standard error), SMD (standardized mean difference) calculated as Hedge’s *g*.
