# Supplementary material for: Lipopolysaccharide‐Induced Bone Loss in Rodent Models: A Systematic Review and Meta‐Analysis
Source: J Bone Miner Res. 2022 Dec 5;38(1):198–213. doi: 10.1002/jbmr.4740 (PMC10107812; doi:10.1002/jbmr.4740)
Supplement: Supplementary file 5 — Table S1. Full systematic search strategy by database. [file JBMR-38-198-s003.docx]

**Supplementary Table 1.** Full systematic search strategy by database.

|  | MEDLINE (OVID) | Embase (OVID) | Web of Science Core Collection (Web of Science) | CINAHL (EBSCO) | Dissertations & Theses Global (PROQUEST) |
| --- | --- | --- | --- | --- | --- |
| **Concept 1:**  Lipopolysaccharide | **Headings:**  exp Endotoxins/  exp Lipopolysaccharides/  **Keywords and phrases:**  (Lipopolysaccharide* or LPS or Lipoglycans or Lipid A or O Antigen* or Endotoxin*).mp. | **Headings:**  Exp Endotoxin/  Exp Lipopolysaccharide/  **Keywords and phrases:**  (Lipopolysaccharide* or LPS or Lipoglycans or Lipid A or O Antigen* or Endotoxin*).mp. | **Headings:** X    **Keywords and phrases:**  (TS=(Lipopolysaccharide* or LPS or Lipoglycans or “Lipid A” or “O Antigen*” or Endotoxin*)) | **Headings:**  (MH "Endotoxins+")  (MH “Lipopolysaccharides”)  **Keywords and phrases:**  (Lipopolysaccharide* or LPS or Lipoglycans or “Lipid A” or “O Antigen*” or Endotoxin*) | **Headings: X**    **Keywords and phrases:**  (Lipopolysaccharide* or LPS or Lipoglycans or “Lipid A” or “O Antigen*” or Endotoxin*) |
| **Concept 2:**  Bone | **Headings:**  *”bone and bones”/ or exp femur/ *leg bones/ or *fibula/ or *tibia/ or exp arm bones/ or *diaphysis/ or exp epiphysis/ or *spine/ or *lumbar vertebra/    **Keywords and phrases:**  (Bone tissue* or Bone density or Bone loss or Bone Microarchitect* or Bone Histomorphometr* or Cancellous bone* or Spongy bone* or Trabecular bone* or Cortical bone* or Cortical thickness or Compact bone* or Bone volume or Trabecular number or Trabecular thickness or Trabecular separation or Bone Strength or Mineral Apposition Rate).mp.  Bone adj2 mineral  Bone adj2 structure* | **Headings:** *bone/ or exp femur/ or exp fibula/ or exp tibia/ or exp arm bone/ or exp diaphysis/ or exp epiphysis/ or *epiphysis plate/ or *spine/ or exp lumbar spine/ or *vertebra/ or *vertebra body/  **Keywords and phrases:**  (Bone tissue* or Bone density or Bone loss or Bone Microarchitect* or Bone Histophometr* or Cancellous bone* or Spongy bone* or Trabecular bone* or Cortical bone* or Cortical thickness or Compact bone* or Bone volume or Trabecular number or Trabecular thickness or Trabecular separation or Bone Strength or Mineral Apposition Rate) .mp.  Bone adj2 mineral  Bone adj2 structure* | **Headings: X**  **Keywords and phrases:**  (“Bone tissue*” or “Bone density” or “Bone loss” or “Bone Microarchitect*” or “Bone Histomorphometr*” or “Cancellous bone*” or “Spongy bone*” or “Trabecular bone*” or “Cortical bone*” or “Cortical thickness” or “Compact bone*” or “Bone volume” or “Trabecular number” or “Trabecular thickness” or “Trabecular separation” or “Bone Strength” or “Mineral Apposition Rate”) OR (“Bone NEAR/2 mineral”) OR (“Bone NEAR/2 structure*”) | **Headings:** (MH "Bone and Bones") OR (MH "Humerus") OR (MH "Radius") OR (MH "Ulna") OR (MH "Cancellous Bone") OR (MH "Cortical Bone") OR (MH "Diaphyses") OR (MH "Epiphyses") OR (MH "Leg Bones") OR (MH "Femur+") OR (MH "Fibula") OR (MH "Tibia") OR (MH "Spine") OR (MH "Lumbar Vertebrae")  **Keywords and phrases:**  (“Bone tissue*” or “Bone density” or “Bone loss” or “Bone Microarchitect*” or “Bone Histomorphometr*” or “Cancellous bone*” or “Spongy bone*” or “Trabecular bone*” or “Cortical bone*” or “Cortical thickness” or “Compact bone*” or “Bone volume” or “Trabecular number” or “Trabecular thickness” or “Trabecular separation” or “Bone Strength” or “Mineral Apposition Rate”)  OR (Bone N2 mineral) OR (Bone N2 structure*) | **Headings: X**    **Keywords and phrases:**  (“Bone tissue*” or “Bone density” or “Bone loss” or “Bone Microarchitect*” or “Bone Histomorphometr*” or “Cancellous bone*” or “Spongy bone*” or “Trabecular bone*” or “Cortical bone*” or “Cortical thickness” or “Compact bone*” or “Bone volume” or “Trabecular number” or “Trabecular thickness” or “Trabecular separation” or “Bone Strength” or “Mineral Apposition Rate”) OR (Bone Near/2 mineral) OR (Bone Near/2 structure*) |
| **Concept 3:**  Rodent Models | **Headings:**  Exp rodentia/  **Keywords and phrases:**  (Rodent* or Mouse or Mice or Mus or Rat or Rats or Guinea pig* or Cricetinae or Hamster*).mp. | **Headings:**  Exp/ rodent  **Keywords and phrases:**  (Rodent* or Mouse or Mice or Mus or Rat or Rats or Guinea pig* or Cricetinae or Hamster*).mp. | **Headings: X**    **Keywords and phrases:**  Rodent* or Mouse or Mice or Mus or Rat or Rats or “Guinea pig*” or Cricetinae or Hamster* | **Headings:**  (MH Rodents+)  **Keywords and phrases:**  (Rodent* or Mouse or Mice or Mus or Rat or Rats or “Guinea pig*” or Cricetinae or Hamster*) | **Headings:**  **Keywords and phrases:**  Rodent* or Mouse or Mice or Mus or Rat or Rats or “Guinea pig*” or Cricetinae or Hamster* |
