# Supplementary material for: Lipopolysaccharide‐Induced Bone Loss in Rodent Models: A Systematic Review and Meta‐Analysis
Source: J Bone Miner Res. 2022 Dec 5;38(1):198–213. doi: 10.1002/jbmr.4740 (PMC10107812; doi:10.1002/jbmr.4740)
Supplement: Supplementary file 6 — Table S2. Summary of risk of bias assessment guidelines. [file JBMR-38-198-s005.docx]

**Supplementary Table 3.** Systematic review summary of study characteristics. Sample size represents the number of studies.

* Calculations for LPS dose are based on a 30 g mouse and a 300 g rat.

|  | **Systematic Review of Intervention Studies using LPS** | | |
| --- | --- | --- | --- |
|  | **< 2 weeks LPS intervention** | **> 2 weeks LPS**  **intervention** | |
| **LPS intervention** | | | |
| Delivery method | I.P. injection | I.P. injection | Slow- release pellets |
| Dose  Absolute | N/A | N/A | Mice  1.33 – 1.5 μg/d  Rat  33.3 μg/d |
| Dose  Relative | Mice  1250 – 20000 μg/kg/injection  *Equivalent to 37.5 – 600 μg/injection**  Rat  5000 μg/kg  *Equivalent to 1500 μg/injection** | Mice  1000 – 25000 μg/kg/injection  *Equivalent to 30 – 750 μg/injection**  Rat N/A | Mice  10 – 100 μg/kg/d  *Equivalent to 0.3 – 3 μg/d**  Rat N/A |
| Delivery schedule | 2-3 injections throughout study period | 4 daily injections throughout study period | Continuous |
| Duration | 2- 14 days | 16 - 28 days | 3-13 weeks |
| **Animal characteristics** | | | |
| Age | 4 - 12 weeks | 5 – 56 weeks | |
| Sex | Male (n = 58)  Female (n = 5)  Unspecified (n = 28) | Male (n = 6)  Female (n = 11)  Unspecified (n = 2) | |
| Species | Mice (n = 89)  Rats (n=2) | Mice (n = 14)  Rats (n = 5) | |
| Strain | ICR, C57BL/6J, BALB/c, ddY, DBA/1J, Sprague-Dawley | C57BL/6J, DBA/2, WT  Sprague-Dawley | |
