# Supplementary material for: Lipopolysaccharide‐Induced Bone Loss in Rodent Models: A Systematic Review and Meta‐Analysis
Source: J Bone Miner Res. 2022 Dec 5;38(1):198–213. doi: 10.1002/jbmr.4740 (PMC10107812; doi:10.1002/jbmr.4740)
Supplement: Supplementary file 7 — Table S3. Systematic review summary of study characteristics. Sample size represents number of studies. [file JBMR-38-198-s001.docx]

**Supplementary Table 2.** Summary of risk of bias assessment guidelines.

|  | Yes | No | Unclear |
| --- | --- | --- | --- |
| Selection bias | | | |
| Sequence generation | Description of the random component in the allocation sequence generation. | No mention of sequence allocation. | When randomization was mentioned but not specified how the allocation sequence was generated. |
| Baseline characteristics | When individual animals were equally distributed by body weight between study groups at baseline. | No body weight data was reported. | When baseline body weight was reported but distribution between study groups was not mentioned. |
| Allocation concealment | When sufficient detail was provided on the method for concealing animal intervention allocation. | N/A | When no information was provided. |
| Performance bias | | | |
| Random housing | When animals were randomly housed during the study. | N/A | When no information was provided. |
| Blinding | When sufficient detail regarding the measures taken to blind the caregivers and researchers from the animal intervention groups. | N/A | When no information was provided. |
| Detection bias | | | |
| Random outcome assessment | When animals were randomly selected for outcome assessment. | N/A | When no information was provided. |
| Blinding | When researchers were blinded during analysis from animal intervention groups. | N/A | When no information was provided. |
| Attrition bias | | | |
| Incomplete outcome data | When the sample size between the methods and results was clearly explained. | When the sample size did not match between the methods and results with no explanation. | When the sample size matched between the methods and results with no explanation. |
| Reporting bias | | | |
| Selective outcome reporting | When details regarding outcome reporting were selected and scored. | N/A | When no explanation was included. |
| Other | | | |
| Other sources of bias | When any other potential sources of bias were discussed in sufficient detail. | N/A | When no other information was discussed. |
